# Memory Accessibility and Medical Decision-Making for Significant Others: The Role of Socially Shared Retrieval-Induced Forgetting

**DOI:** 10.3389/fnbeh.2013.00072

**Published:** 2013-06-14

**Authors:** Dora Coman, Alin Coman, William Hirst

**Affiliations:** ^1^Department of Psychology, New School for Social Research, The New School, New York, NY, USA; ^2^Department of Psychology, Princeton University, Princeton, NJ, USA

**Keywords:** memory accessibility, retrieval-induced forgetting, medical information

## Abstract

Medical decisions will often entail a broad search for relevant information. No sources alone may offer a complete picture, and many may be selective in their presentation. This selectivity may induce forgetting for previously learned material, thereby adversely affecting medical decision-making. In the study phase of two experiments, participants learned information about a fictitious disease and advantages and disadvantages of four treatment options. In the subsequent practice phase, they read a pamphlet selectively presenting either relevant (Experiment 1) or irrelevant (Experiment 2) advantages or disadvantages. A final cued recall followed and, in Experiment 2, a decision as to the best treatment for a patient. Not only did reading the pamphlet induce forgetting for related and unmentioned information, the induced forgetting adversely affected decision-making. The research provides a cautionary note about the risks of searching through selectively presented information when making a medical decision.

## Introduction

Medical decisions, like many other kinds of decisions, will often entail a broad search for a wide range of relevant information. When deciding which treatment option to pursue, people might visit one or more doctor(s), scan the Internet, talk to friends and acquaintances, and acquire and carefully peruse relevant brochures and other printed material. Given the commercial and often biased nature of many sources of information, as well as time constraints faced by the investigator, the received information might highlight some facts, while limiting easy access to other, equally relevant ones (Simon, 1985; Gigerenzer and Goldstein, 1996). For instance, the Pfizer website for Lipitor (Lipitor Official Website, 2006) (http://www.lipitor.com) describes the drug’s side effects and precautions only in a side bar or in a manner that demands that the viewer scrolls down to the bottom of the web page. We are interested here in how selective presentation of information affects the subsequent accessibility of the “unmentioned” items. Moreover, we want to explore whether any shift in accessibility potentially influences medical decisions.

In many instances, medical decisions are best characterized as based on memory for the “gist” of acquired information (Reyna and Lloyd, 2006). For instance, people often fail to remember the exact figures provided about the risk of a medical procedure, even though they may remember the “general” pattern (Reyna and Hamilton, 2001). Providing the gist about the relative risk of different treatment options can be more effective than providing precise information (for a review, see Reyna, 2008).

In some situations, however, access to precise information may be desirable for the medical decision-maker. When deciding what citrus fruit to have at breakfast, decision-makers may be at an advantage if they know precisely that navel and Valencia oranges are allowable if taking Lipitor, but not Cara Cara oranges or grapefruit. Knowing simply that some citrus fruits are allowable may not be sufficient. In such situations, knowing and accessing specific information might become critical for making effective judgments and decisions (see Lichtenstein et al., 1978; Lynch and Srull, 1982; Feldman and Lynch, 1988; Johnson et al., 2007; see also Tversky and Kahneman, 1973; Menon and Raghubir, 2003).

The present study, then, addresses two issues: how does selective exposure to relevant medical information affect mnemonic accessibility? And in instances in which precise information is needed, does any shift in mnemonic accessibility influence medical decision-making? We focus on situations in which the decision-maker is first exposed to information on the treatments suitable for a disease and then re-exposed to a selective rendering of those treatments, as might be the case when a patient turns to the Internet to follow-up on the discussion they had with their doctor. With such re-exposures, even if time constraints do not prevent a complete and exhaustive search, there might nevertheless be selectivity, in that, as noted, all the relevant information encountered initially might not easily be found during an Internet search. Our interest is not in the practice effects one might expect to find with re-exposure, but in the forgetting that might occur when information goes unmentioned.

There are several reasons why forgetting might occur when previously known information goes unmentioned. First, the unmentioned information might decay if not rehearsed (Wixted, 2004). Second, and more critical for the present study, individuals might be *induced* to forget the unmentioned information if it is related to mentioned information. As the large literature on retrieval-induced forgetting (RIF) suggests, when people selectively practice previously studied material, they are more likely to forget unpracticed memories related to the practiced material than unpracticed, unrelated memories (Anderson et al., 1994).

The standard RIF paradigm includes three experimental phases: a study phase, a selective practice phase, and a final recall phase. In the study phase participants study category-exemplars pairs such as: *fruit-apple*, *fruit-banana*, *clothes-dress*, *clothes-shirt*. A selective practice phase follows where participants are instructed to complete stems for half of the exemplars from half of the categories: *fruit-a*____, but not *fruit-banana*, nor any of the *clothes* items. This selective practice creates three types of items, depending on the retrieval status of each studied item: (1) items that were selectively practiced (Rp+: *fruit-apple*), (2) items that were unpracticed, but related to the practiced ones (Rp−: *fruit-banana*), and (3) items that were unpracticed and unrelated to those practiced (Nrp: *clothes-dress*; *clothes-shirt*). Finally, participants are asked to recall all exemplars from all categories presented in the study phase. The recall proportion measured in the final recall phase reveals both a practice effect for practiced items (that is, the recall proportion of Rp+ items larger than the recall proportion of Nrp items), but, more importantly for this paper, RIF is observed for the unpracticed, related material (that is, the recall proportion of Rp− items smaller than the recall proportion of Nrp items). If RIF were merely a matter of decay, the unpracticed memories related to the retrieved materials should be forgotten at the same rate as unpracticed memories unrelated to the retrieved material. In order to account for the observed RIF pattern, many researchers have argued that the retrieval of a desired memory will produce response competition from related memories, which must be inhibited if retrieval of the desired memory is to be successful (for a review, see Anderson and Levy, 2007; Storm and Levy, 2012). This inhibition lingers, producing the pattern of forgetting associated with RIF (for an alternative account, see Dodd et al., 2006).

Retrieval-induced forgetting is relevant to our present concerns because it can occur not only when probed individuals themselves selectively and overtly remember (within-individual RIF, or WI-RIF), but also when probed individuals attend to others remembering (socially shared RIF, SS-RIF; Cuc et al., 2007; Coman et al., 2009; Stone et al., 2010; Coman and Hirst, 2012; Hirst and Echterhoff, 2012). Hirst and his colleagues claim that SS-RIF occurs because attendees concurrently retrieve with the rememberer, and, as a result, find themselves also selectively remembering. In some instances of SS-RIF, the source of the memory can be physically present, as when a listener monitors the speaker for accuracy in a conversation. In other instances, it can be implied, as when someone reads written material. For reading, the source of the memory is the “author” of the material. SS-RIF differs from WI-RIF because in the latter case, the experimenter instructs participants to retrieve particular memories. Speakers or authors, when discussing the past, are, by definition, retrieving memories. Their listeners or readers, however, are not obligated to retrieve a memory along with the speaker or author. If they retrieve overtly, they are no longer a listener, but a speaker. If they retrieve covertly along with the speaker, it is entirely a choice that they alone have made. What is perhaps surprising is that listeners and readers appear to make the effort to concurrently, covertly retrieve in many instances, thereby manifesting SS-RIF.

Studies of SS-RIF, especially those involving written material, would suggest that selective practice^1^ of medical information might induce forgetting for unpracticed, but related information. The result would be a hierarchy of accessibility, with the practiced information most accessible, the unpracticed, and unrelated to the practiced information moderately accessible, and the unpracticed information related to the practiced information least accessible. Our claim is that these differences in accessibility have consequences for the final medical decision. For instance, a person with back pain may initially learn about the advantages and disadvantages of two treatments, steroids and acupuncture, but may encounter only the advantages of the steroid treatment as they continue their search. They may also fail to encounter any information about acupuncture, given, perhaps, its “alternative-medicine” status. If SS-RIF is at work, they should in the end have more difficulty remembering the disadvantages of steroid treatment than the disadvantages of acupuncture. This difference in accessibility could affect their decision about which treatment to pursue.

The relation between RIF and decision-making has proven difficult to establish. Storm et al. (2005), for instance, found that RIF for positive and negative attributes of target individuals did not affect participants’ impressions of the targets, at least as measured by likeability ratings. On the other hand, Iglesias-Parro and Gomez-Ariza (2006) found that the selective practice of previously studied material about job candidates influenced participants’ employment judgments, but only in certain circumstances.

In the present study, we examined for the first time the relation between SS-RIF and medical decision-making. In Experiment 1, we determined whether we could find SS-RIF for medical information. As we noted, when reading, simultaneous retrieval of relevant memories is optional. SS-RIF will only occur if covert concurrent retrieval occurs (Cuc et al., 2007). We therefore wanted to determine, before proceeding to our questions about RIF and decision-making, whether selectively presented medically relevant material induced forgetting in previously learned medical information. Participants first learned about a fictitious disease, Wheeler’s syndrome, including the advantages and disadvantages of treatment options. We chose to use as our stimulus material a fictitious disease because we wanted to avoid any effect prior knowledge might have had on memory. Other researchers have employed a similar strategy for similar reasons (e.g., John and Fischhoff, 2010). We sought to make the disease as realistic as possible by modeling it on known diseases. Participants then read a brochure that presented information selectively. The pamphlet discussed some treatments, while ignoring others, and stressed either advantages or disadvantages for these discussed treatments. A final recall test followed. The results of Experiment 1 should establish that the act of reading selectively presented information can induce forgetting in initially encoded memories that are related to the practiced information.

Experiment 2 explored the more critical issue of whether the SS-RIF observed in Experiment 1 will influence decision-making. In order to understand this dynamic, we added a final decision-making task at the end of the experiment. Importantly, for Experiment 2 we changed the design of the material so as to exclude the possibility that retrieval practice effects might account for the final decision, thereby limiting any possible mnemonic effects to SS-RIF.

In both experiments, participants are asked to imagine that they are helping a friend make a decision. We could have asked participants to imagine that they had the described disease, but we reasoned that it might seem more realistic to participants to imagine that they were helping a friend with the described disease make a decision. People often experience medical decisions as stressful (Loewenstein, 2005; Luce, 2005) and, thus, besides receiving a doctor’s opinion, they might consult with family and friends and rely on them for gathering treatment relevant information (Srirangam et al., 2003; Boehmer and Babayan, 2005).

## Experiment 1

### Method

#### Participants

Twenty-four undergraduate New School students received research credits for experimental participation. Data from two participants was discarded because in the debriefing phase of the study, they reported skepticism about the existence of the disease.

#### Materials

We constructed a 180-word description of Wheeler’s syndrome. We made the information included in the description as plausible as possible by keeping close to syndrome descriptions in The Merck Manual, a widely used manual for diagnosis and treatments of medical disorders (Beers et al., 2006). Its definition, its prevalence and incidence were presented on one PowerPoint slide and its stages on another slide. On the third slide, participants learned information about Laura, whom participants were instructed to view as their fictional best friend. Four treatment options were then presented, in a random order, in a series of PowerPoint slides, one option per slide. On each slide, there was the name of the treatment, in bold type (e.g., **Propionic**, **Metabotropic**, etc.) and immediately below three advantages and three disadvantages, although they were not labeled as such. In fact, their benefits or costs were specific to the fictional friend the participants had learned about. For instance, the assertion that one treatment could be taken along with antacid medication was viewed as an advantage because Laura took antacid medication. The advantages were always framed “positively,” e.g., “The treatment can be taken by people with stomach problems.” (Laura has stomach problems.) Disadvantages were framed “negatively,” as in “The treatment causes side effects for people with kidney problems.” (Laura has kidney problems.) The form of the statement (whether it was an advantage, and thus positively framed or a disadvantage, and thus negatively framed) will be treated as a Valence factor in this experiment. Three evaluators analyzed each item of information included in each of the four treatments on the following dimensions: (a) salience of the items (e.g., whether some items were more salient and more likely to be easily remembered compared to others), and (b) relevance of items for Laura’s case (e.g., whether some items were more relevant to her medical profile compared to others). Items evaluated as more salient and/or more connected to Laura’s medical profile were discarded and replaced by new items. This pilot work indicated that participants should find each treatment – with its respective advantages and disadvantages – equally memorable and relevant for Laura’s case. The advantages always preceded the disadvantages, but were otherwise randomly presented on the slide.

We chose to present advantages first for two related reasons. First, we wanted to avoid possible framing effects. Specifically, people tend to be more receptive at selecting treatment options when they are presented in a positive frame (Moxey et al., 2003). If we had varied the order, we might have built in biases that would have been difficult to compensate for, even with appropriate counterbalancing. Second, our decision reflects what we believe is the decision of many authors of medical brochures and Internet sites. A survey of medical websites indicated that most begin a description of a treatment with advantages, leaving the disadvantages to last. Indeed, it would seem odd to most people to begin a description of a treatment by articulating its disadvantages.

“Additional practice” was given with a brochure about Wheeler’s syndrome. It contained a title page, which announced the disease’s name and the sponsoring institution. The second page contained the same description of the disease presented during the original study phase. The third, and last, page described the treatment options, but selectively and in a random order. The top of the third page contained instructions asking the reader to indicate in the brochure whether each item under the treatment labels below would best be viewed as an advantage or a disadvantage. The treatments then followed. Two of the four initially presented treatment alternatives were presented, and for both of these treatments, either only the advantages or only the disadvantages were discussed. Participants were asked to indicate for each of the six statements whether they could be viewed as either an advantage or a disadvantage. These subjective judgments conformed to our classification 99% of the time. Which of the treatments were practiced was counterbalanced. To this end, we created four brochures.

This selective presentation of treatments with advantages or disadvantages allowed us to create Rp+, Rp−, and Nrp items. (The advantages or disadvantages presented in the brochure were Rp+; the non-mentioned advantages or disadvantages that were part of the same treatments with those mentioned were Rp−; and the advantages and disadvantages of the unmentioned treatments, Nrp.) *Valence* will refer to whether the Rp+ items were advantages (Positive Valence) or disadvantages (Negative Valence). Thus, page 3 contained two treatment options, with a total of either six advantages or six disadvantages. If Rp+ items were advantages, the Rp− items were disadvantages, and vice versa.

As to the description of Laura, it consisted of a 391-word profile. The inclusion of a target individual allowed the participant to understand the advantages and disadvantages as they apply to Laura. The profile indicated that Laura was recently diagnosed with stage II Wheeler’s Syndrome and included age, personal information, as well as medical history.

#### Design and procedure

**Figure 1:**
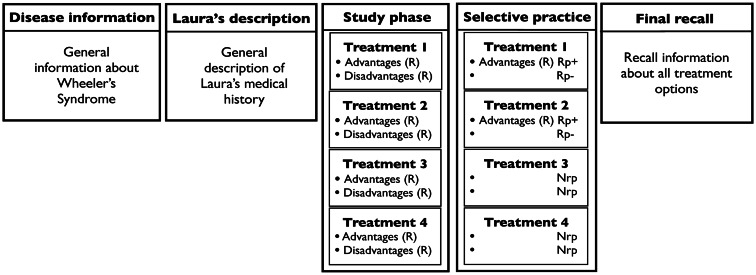
**Phases of Experiment 1, for the Positive Valence Condition**. In the Study and Selective practice phases, R stands for relevant information; Rp+ for retrieval practice plus; Rp− for retrieval practice minus, and Nrp for no retrieval practice. In the Negative Valence condition, participants selectively practiced relevant disadvantages for two of the four treatments.

All material was presented on an iMac computer. Participants first read the PowerPoint slides describing Wheeler’s syndrome, each presented for 80 s. Then, Laura’s profile appeared on the computer screen for 100 s. The screen then turned blank and participants were given 15 four-item forced choice recognition probes to test whether they remembered the information about Laura. The experimenter corrected any errors in the recognition test in front of the participants. The presentation of the four treatment alternatives commenced. Each treatment slide appeared on the computer screen for 45 s. Participants were then asked to peruse the brochure, which constituted the selective practice phase. They were given 7 min to do so. Finally, participants completed a cued recall for the advantages and disadvantages of the four treatments, with the name of the treatment serving as cue. There were 5 min of distraction between each phases of the experiment (see Figure 1 for a summary of experimental phases for Experiment 1).

### Results and discussion

**Figure 2:**
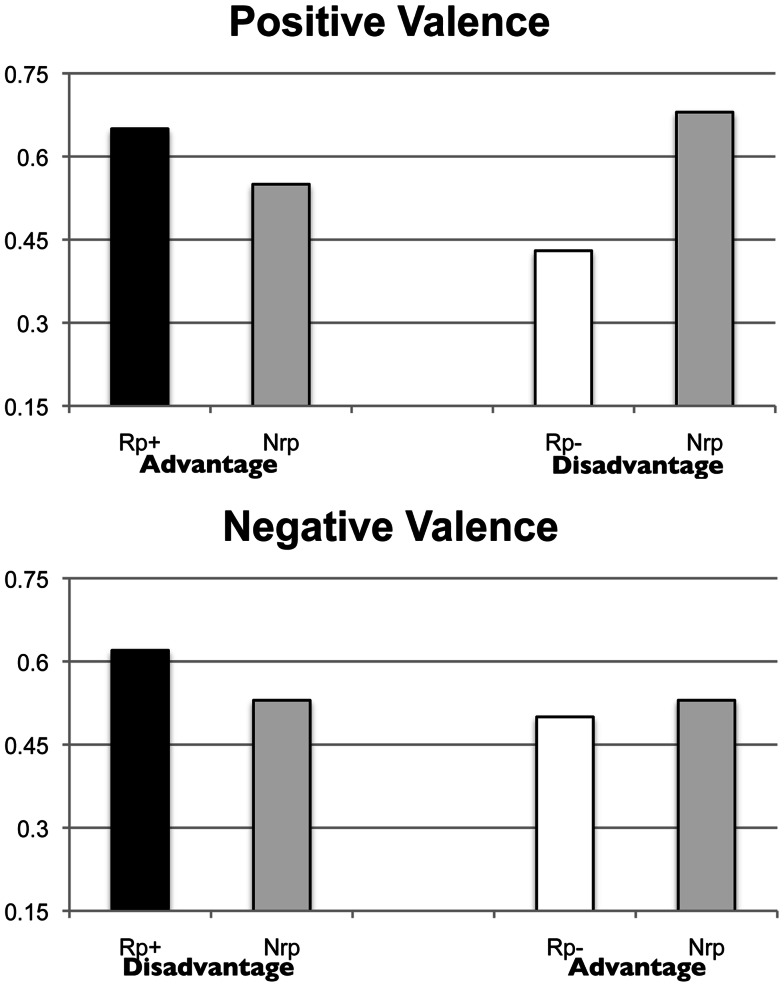
**Recall proportion for Rp+/Nrp and Rp−/Nrp items in Experiment 1, for the Positive Valence Condition (advantages received selective practice) (on the top), and the Negative Valence Condition (disadvantages received selective practice) (on the bottom)**. In the Positive Valence Condition, we compared Rp+ items (advantages) with Nrp advantage items, and Rp− items (disadvantages) with Nrp disadvantage items. Similarly, in the Negative Valence Condition, we compared Rp+ items (disadvantages) with Nrp disadvantage items, and Rp− items (advantages) with Nrp advantage items.

To examine the effect of reading the brochure on memory accessibility, we first undertook two repeated measures analyses of variance (ANOVA), one for the practice effect (Rp+ > Nrp) and another for the induced forgetting effect (Nrp > Rp−). For the practice effect, when the advantages were mentioned in the brochure, we compared the proportion of the remembered Rp+ items out of the total number of Rp+ items with the proportion of remembered advantages of the Nrp treatments out of the total number of advantages of Nrp treatments (thereby comparing advantage with advantage in the final recall test). Similarly, for the induced forgetting effect, when the advantages were mentioned in the brochure we compared the proportion of the remembered Rp− items (related but unmentioned disadvantages) with the proportion of remembered disadvantages of the Nrp treatments (allowing us to compare disadvantages with disadvantages in the final recall). A similar comparison procedure was followed for both the practice and induced forgetting effects when disadvantages, instead of advantages, were practiced (see Figure 2).

For each ANOVA, there was one between-subject factor, Valence (whether advantages or disadvantages were practiced, we will use the terms *positive* and *negative*, respectively, to refer to the two) and one within-subject factor, Retrieval Type (Rp+ vs. Nrp or Rp− vs. Nrp). For the practice effect, we failed to find any significant main effects: Retrieval Type, *F*(1, 18) = 2.26, *p* = 0.15, $\eta_{p}^{2}=0.11$, and Valence, *F*(1, 18) = 0.14, *p* = 0.72, $\eta_{p}^{2}=0.00$. Nor did we find a significant interaction, *F*(1, 18) = 0.02, *p* = 0.89, $\eta_{p}^{2}=0.00$. As for the presence of SS-RIF, our main interest, we found a significant main effect for Retrieval Type, *F*(1, 18) = 4.23, *p* < 0.05, $\eta_{p}^{2}=0.19$, but not for Valence, *F*(1, 18) = 0.34, *p* = 0.57, $\eta_{p}^{2}=0.02$. The interaction was also not significant, *F*(1, 18) = 2.45, *p* = 0.14, $\eta_{p}^{2}=0.12$. We are not the first to find RIF in the absence of a practice effect (e.g., Storm et al., 2006). There is no *a priori* reason why the two must be connected in that they may involve different mechanisms. For instance, practice effects could involve the strengthening of a trace; RIF, the inhibition of a related trace. For SS-RIF, it is the retrieval of an item that triggers the processes that lead to induced forgetting, not the strengthening of the trace associated with the retrieved item (Anderson et al., 2000).

## Experiment 2

Experiment 1 demonstrated that reading selectively presented medical material induced forgetting for unmentioned, but related information. Can this SS-RIF influence subsequent medical decisions? Of course, a practice effect could also affect decision-making by making the practiced items more accessible when the final decision is made. The experimental design of Experiment 1 does not allow us to disentangle easily practice and SS-RIF effects on a subsequent treatment decision. Inasmuch as our interest is the impact of SS-RIF on decision-making, in Experiment 2, we designed the material so that any contribution of a practice effect to the final treatment decision became irrelevant. Specifically, the selective practice brochure was rewritten so that it only covered material irrelevant to Laura’s case (e.g., “It can be taken by people who are allergic to aspirin,” is irrelevant because Laura is not allergic to aspirin). The related, unmentioned information was, however, relevant (e.g., “It may have side effects for patients who have kidney problems,” is relevant because Laura has kidney problems). We wanted to examine whether practicing irrelevant material could have the potential to decrease the accessibility of related, unmentioned information, which could, in turn, affect decision-making concerning Laura’s treatment.

### Method

#### Participants

Forty-eight graduate and undergraduate New School students participated in the experiment for class credit. Participants were divided evenly between the Test-Present and Test-Absent conditions and within these divisions, between the Positive and Negative Valence conditions.

#### Materials and design

The description of Wheeler’s syndrome was the same as in Experiment 1. Similarly, each of the four treatments presented in the study phase included three advantages and three disadvantages, for a total of six items per treatment. Unlike Experiment 1, each advantage or disadvantage appeared on its own PowerPoint slide. Although in Experiment 1 all the advantages or disadvantages were relevant to Laura’s case, in Experiment 2, a statement (be it an advantage or a disadvantage) could be relevant or irrelevant. We constructed four types of statements: *irrelevant advantage*, *irrelevant disadvantage*, *relevant advantage*, *and relevant disadvantage*. The statement “The treatment is recommended to people with Lupus” was *irrelevant* because Laura doesn’t have Lupus and an *advantage* because it contains an inclusion criterion. A variant of the above statement – “The treatment is not recommended to people with Lupus,” – was an *irrelevant disadvantage* because, although Laura doesn’t have Lupus, the statement involves an exclusion criterion. On the other hand, the statement “The treatment can be taken by people with stomach problems” is both *relevant* (Laura has stomach problems) and an *advantage*, in that it contains an inclusion criterion. Finally, the statement “The treatment cannot be taken by people with stomach problems” is a *relevant disadvantage* because it contains an exclusion criterion. In what follows, we use the terms *positive* and *advantage*, as well as *negative* and *disadvantage*, interchangeably.

As for the brochure used in the practice phase, as in Experiment 1, only two treatments were included, with only the three irrelevant statements mentioned for each treatment. There were four brochures. Two of the brochures mentioned only the irrelevant advantages of the two treatments (constituting one set of brochures) and were given to participants in the Positive Valence Condition. The other two mentioned only the irrelevant disadvantages of the two treatments (constituting the second set) and were given to participants in the Negative Valence Condition. For each of the sets of the two brochures, which treatments were mentioned was counterbalanced, so that in one brochure Treatments 1 and 2 were mentioned, while Treatments 3 and 4 went unmentioned. For the other brochure in a set, Treatments 3 and 4 were mentioned, while Treatments 1 and 2 went unmentioned. Participants did not know that the mentioned information was irrelevant, inasmuch as, in Experiment 2, Laura’s profile was presented toward the end of the experiment, in the decision-making phase, and the information is relevant or irrelevant only in the context of Laura’s profile. The brochure had the same format as the brochure used in Experiment 1. Just as in Experiment 1, participants were asked to indicate for each of the six statements in the brochure whether they can be viewed as an advantage or a disadvantage. These subjective judgments conformed to our classification 94% of the time.

As to Laura’s profile, it was similar to what was used in Experiment 1, with only slight stylistic changes. Unlike in Experiment 1, the profile was presented at the end of the experiment, when participants had to make a treatment decision, because we wanted to ensure that participants carefully read the material during the practice phase, which they might not have if they deemed it irrelevant. People will often search for information on a disease of a friend before they know the details of her medical history.

#### Design and procedure

Participants first read the PowerPoint presentation about Wheeler’s syndrome. They then read a presentation of the four treatment alternatives. Each item from each treatment was presented on a separate slide. For counterbalancing, half of the participants were exposed to four treatments containing three irrelevant advantages and three relevant disadvantages. In a mirror image pattern, the other half of the participants was presented with four treatments containing three irrelevant disadvantages and three relevant advantages.

**Figure 3:**
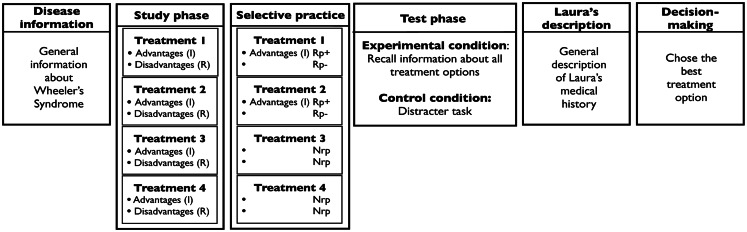
**Phases of Experiment 2, for the Positive Valence condition**. In the Study and Selective practice phases, R stands for relevant information; I stands for irrelevant information; Rp+ for retrieval practice plus; Rp− for retrieval practice minus, and Nrp for no retrieval practice. In the Negative Valence condition, participants studied irrelevant disadvantages (the negatively stated version of irrelevant advantages) and relevant advantages (the positively stated version of relevant disadvantages) for each treatment, and selectively practiced irrelevant disadvantages for two of the four treatments.

After a distracter task, participants were given the brochure and asked to read it carefully. Half of the participants were given the two types of brochures designed for the Positive Valence Condition; the other half, the brochures designed for the Negative Valence Condition. Participants were given 7 min to read the brochure. As in Experiment 1, after another distracter task, in the Test-Present condition, participants completed a final recall test. They were given 10 min to complete it. The Test-Absent condition dropped the final recall test and extended the distracter task by 10 min. This control eliminated any potential effect that the final recall might have on decision-making. After the recall test or the extended distracter, participants received a hard copy of Laura’s profile and were asked to choose the best treatment alternative for their friend’s medical condition. They had as long as they wished to make a decision. See Figure 3 for a summary of experimental phases of Experiment 2.

### Results and discussion

As in Experiment 1, we first wanted to establish whether reading the brochure resulted in practice and SS-RIF effects. Here we examined the proportion of Rp+, Rp−, and Nrp items remembered in the final recall test of the Test-Present (experimental) condition. We then explored the relation between memory accessibility and decision-making.

#### Retrieval effects

**Figure 4:**
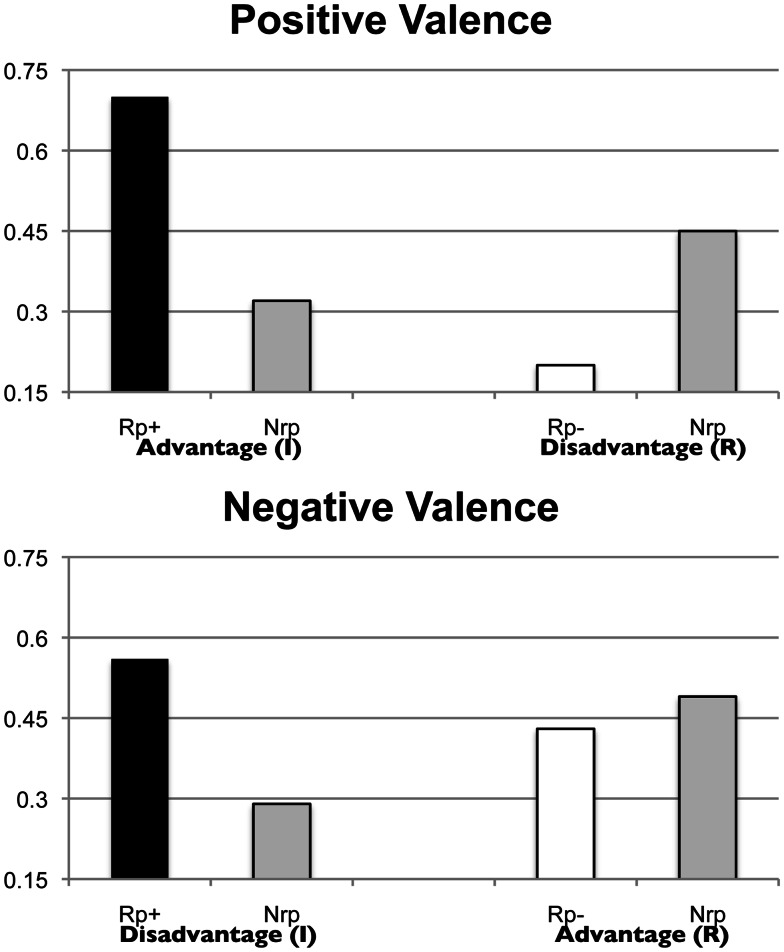
**Recall proportion for Rp+/Nrp and Rp−/Nrp items in Experiment 2, for the Positive Valence Condition (irrelevant advantages received selective practice) (on the top), and the Negative Valence Condition (irrelevant disadvantages received selective practice) (on the bottom)**. R stands for relevant, and I stands for irrelevant. In the Positive Valence Condition, we compared Rp+ items (irrelevant advantages) with Nrp advantage items, and Rp− items (irrelevant disadvantages) with Nrp disadvantage items. Similarly, in the Negative Valence Condition, we compared Rp+ items (irrelevant disadvantages) with Nrp disadvantage items, and Rp− items (irrelevant advantages) with Nrp advantage items.

We undertook two repeated measures ANOVA with Valence (whether irrelevant advantages or irrelevant disadvantages were practiced) as a between-subject factor, and Retrieval Type (Rp+ vs. Nrp or Rp− vs. Nrp) as a within-subject factor. Just as in Experiment 1, the recall proportion in the final recall test served as the dependent variable. As in Experiment 1, we compared the recall proportion of Rp advantages with the recall proportion of Nrp advantages, and similarly, we compared the recall proportion of Rp disadvantages with the recall proportion of Nrp disadvantages (see Figure 4). For the practice effect, we only found a significant main effect for Retrieval Type, *F*(1, 22) = 22.87, *p* < 0.001, $\eta_{p}^{2}=0.51$. There was no significant main effect for Valence, *F*(1, 22) = 2.22, *p* = 0.15, $\eta_{p}^{2}=0.09$, or for the interaction, *F*(1, 22) = 0.71, *p* = 0.71, $\eta_{p}^{2}=0.03$. That is, unlike Experiment 1, for which we did not find a practice effect, both advantages and disadvantages benefited from additional rehearsal here.

For the induced forgetting effect, we found significant main effects for both Retrieval Type, *F*(1, 22) = 8.61, *p* < 0.01, $\eta_{p}^{2}=0.18$, and Valence, *F*(1, 22) = 4.45, *p* < 0.05, $\eta_{p}^{2}=0.17$. There was no significant effect for the interaction between Retrieval Type and Valence, *F*(1, 22) = 3.48, *p* = 0.08, $\eta_{p}^{2}=0.13$. Importantly, we replicated Experiment 1 by finding SS-RIF.

One explanation for the observed induced forgetting effect is the output interference hypothesis, according to which impairment arises because the recalled Rp+ items interfere with the recall of Rp− items (Anderson and Spellman, 1995). As a test of this possibility, we followed Macrae and Roseveare (2002; see also Barnier et al., 2004) and ranked the Rp+ and Rp− items according to the order in which they appeared in each participant’s final recall, with the lower ranking indicating an earlier recall. We then averaged the rankings for recalled Rp+ and Rp− items. We performed an ANOVA with Retrieval Type (Mean Rank Rp+ vs. Mean Rank Rp−) as a within-subject factor and Valence as a between-subject factor. We found neither significant main effects, nor a significant interaction (all *p*’s > 0.20), which means that the induced forgetting effect is not due to the interference caused by the selective recall of Rp+ items.

#### Decision-making

As to the effect of SS-RIF on the decision about Laura’s treatment, we undertook two separate analyses. In the first one, we classified the decisions as to whether they were consistent or inconsistent with what was assumed to be forgotten after reading the brochure. For example, if participants read a pamphlet about irrelevant advantages of some, but not all treatments, they would subsequently have difficulty accessing relevant, related and unmentioned disadvantages. As a result, according to the SS-RIF model, the disadvantages of those treatments discussed in the brochure should be less accessible than the disadvantages of the unrelated and unmentioned treatments. Inasmuch as the design of our stimulus material ensured that we can discount any influence practice might have on subsequent decision-making, then, in the present example, participants should prefer the mentioned treatments, in that their disadvantages are relatively inaccessible. Along similar lines, when irrelevant disadvantages are practiced, the relevant, related, and unmentioned advantages should subsequently be less accessible than the relevant advantages of the unrelated and unmentioned treatments. In such an instance, participants should prefer the treatments that were not discussed in the brochure because their advantages are more accessible. Inasmuch as we failed to find a difference in the frequency with which participants made a SS-RIF-consistent decision in the Test-Present and Test-Absent conditions, χ^2^(1) = 0.097, *p* = 0.76, we combined the data from these two conditions. Thirty-three out of 48 participants made RIF-consistent decisions, a proportion greater than chance (using a sign test, *p* < 0.02).

The second analysis offered a refinement over the first analysis, in that it examined whether SS-RIF-consistent decisions are more likely as SS-RIF impairment increased. We now focused solely on data from the Test-Present condition. We calculated the size of SS-RIF impairment [(Nrp) – (Rp−)] and the practice effect [(Rp+) – (Nrp)] and then used these scores in a binary logistic regression to test whether these two scores predicted SS-RIF-consistent decisions. We did not expect that the practice effect should make a contribution, inasmuch as the experiment was designed to make practice effects irrelevant. We did expect to see a contribution of SS-RIF impairment. Confirming the hypothesis, a binary logistic regression employing a forward conditional model excluded the practice effect, but included SS-RIF impairment as the only significant predictor, χ^2^(1, *N* = 24) = 5.46, *p* < 0.02; β = 4.45, odds ratio (OR) = 85.65, Wald = 4.01, *p* < 0.05.

## General Discussion

This study had two aims: (1) to determine whether SS-RIF can be observed as a consequence of reading information about a medical treatment, and (2) whether any observed SS-RIF can potentially affect subsequent decision-making. Regarding the first aim, both experiments clearly showed SS-RIF. As we noted in the introduction, the concurrent, covert retrieval underlying SS-RIF is optional. When a person reads about the advantages and disadvantages of treatments in a brochure, they do not need to remember silently any memories they have previously formed about the treatment. The current findings indicate that they do. Moreover, they retrieve what is mentioned in the brochure, but, as the presence of SS-RIF indicates, what they retrieve may inhibit the unmentioned related item. This finding underscores an important cost associated with selective presentation, even for medically relevant information.

As to the second issue addressed in this paper, the results indicate that socially shared RIF is a potential mechanism by which memory accessibility can affect decision-making, at least when the decision depends on the accessibility of precise information. By focusing on induced forgetting, our research adds substantially to previous efforts exploring how memory accessibility critically contributes to medical decision-making by exploring the effects of prior exposure on memory and on subsequent decisions (Redelmeier and Kahneman, 1996; Redelmeier et al., 2003; see also Baines et al., 1990; Erskine et al., 1990). To be sure, practice effects might also contribute, but the design of Experiment 2 eliminated any possible contribution of a practice effect, thereby highlighting the contribution of SS-RIF. How practice and induced forgetting might interact to lead to a final decision in situations where both could play a role will, of course, depend on the relevance of the practiced and forgotten information to the target individual. There is no *a priori* reason to believe that practice effects trump the effects of induced forgetting. For instance, people may be aware of the role of practice and compensate for its influence when making the final decision. They are less likely to be aware of the role of induced forgetting, especially the different pattern of forgetting for unmentioned related over unrelated information. As a result, they might be less likely to adjust for the contribution of SS-RIF.

Several caveats are in order. We need to be cautious about making any general statement, inasmuch as we employed only one example of a disease. However, on the surface, there was nothing extraordinary about our description of Wheeler’s Syndrome. When we asked our participants, in the debriefing phase, to indicate whether Wheeler’s syndrome seems like something a friend might develop under some unfortunate circumstance, 97% responded in the affirmative. Moreover, we cannot be certain whether SS-RIF can drive decision-making if there is a substantial delay between selective practice and the final decision. Some studies suggest that impairment can last no more than 24 h (MacLeod and Macrae, 2001), whereas other studies find RIF after a week (Conroy and Salamon, 2006; Migueles and Garcia-Bajos, 2007; Tandoh and Naka, 2007; Garcia-Bajos et al., 2008; Storm et al., 2012). It is worth noting that even the shorter time frame may still be relevant to decision-making, especially if decision-making occurs in increments, with new or tentative decisions being made as new information is acquired (Johnson et al., 2005; Weber and Johnson, 2006).

In instances when the decision-maker has enough time to consider all advantages and disadvantages while reading a brochure or scanning the internet, selective retrieval might result in retrieval-induced facilitation for the unmentioned and related medical information (Chan et al., 2006). In these situations we suspect that treatment decisions will be driven by the resultant increase in accessibility. Future research should explore the effects of retrieval-induced facilitation, as well as the conditions that might induce facilitation over forgetting.

In the studies presented in this paper advantages always preceded disadvantages. The rationale for this decision was to avoid potential framing effects and to keep treatments’ presentation as ecologically valid as possible. However, further research should also explore whether the order of presentation has a significant impact on RIF.

Finally, in these experiments, participants were asked to make a treatment decision for another individual. Memory accessibility may be impacted differently when the decision is made for self vs. for a significant other. There is some research to support this concern. For instance, decisions made for another individual can induce a higher sense of responsibility in the surrogate, resulting in a preference for more conservative options (Raymark, 2000). This observation, however, does not imply that SS-RIF would only play a role in surrogate decision-making. There is no *a priori* reason why it would also not apply to self-relevant decision.

The research provides a cautionary note about the risks of decision-driven selective retrieval of medical information. When exposed to medical information, like reading a brochure, talking with a doctor, or watching a commercial, the results here suggest that beyond a simple practice effect, selective remembering can induce forgetting for unmentioned, related, and relevant information. This induced forgetting could influence medical decision-making in not necessarily positive ways. When patients and significant others read incomplete information from brochures, Internet entries, or just simply listen to a commercial on the TV, these acts might, in the end, be more detrimental than informative for the decision-making process. A broad implication at the policy level of this research would be for medical providers to include complete and comprehensive information in pamphlets and Internet entries so that patients and their significant others are fully informed when making or advocating for medical decisions.

## Conflict of Interest Statement

The authors declare that the research was conducted in the absence of any commercial or financial relationships that could be construed as a potential conflict of interest.

## Footnotes

^1^Selective practice in this context refers to selectively searching through different sources of medical information. In this search we are re-exposed to parts of previously learned information, while other parts either related or unrelated to the re-exposed one remain unmentioned.
